# Construct Validity of the Dutch Version of the 12-Item Partners in Health Scale: Measuring Patient Self-Management Behaviour and Knowledge in Patients with Chronic Obstructive Pulmonary Disease

**DOI:** 10.1371/journal.pone.0161595

**Published:** 2016-08-26

**Authors:** Anke Lenferink, Tanja Effing, Peter Harvey, Malcolm Battersby, Peter Frith, Wendy van Beurden, Job van der Palen, Muirne C. S. Paap

**Affiliations:** 1 Department of Pulmonary Medicine, Medisch Spectrum Twente, Enschede, The Netherlands; 2 Department of Research Methodology, Measurement, and Data-Analysis, Faculty of Behavioural, Management and Social sciences, University of Twente, Enschede, The Netherlands; 3 School of Medicine, Flinders University, Adelaide, South Australia, Australia; 4 Department of Respiratory Medicine, Repatriation General Hospital, Adelaide, South Australia, Australia; 5 Flinders Human Behaviour and Health Research Unit, Flinders University, Adelaide, Australia; 6 Centre for Educational Measurement at the University of Oslo (CEMO), Faculty of Educational Sciences, University of Oslo, Oslo, Norway; Public Health England, UNITED KINGDOM

## Abstract

**Objective:**

The 12-item Partners in Health scale (PIH) was developed in Australia to measure self-management behaviour and knowledge in patients with chronic diseases, and has undergone several changes. Our aim was to assess the construct validity and reliability of the latest PIH version in Dutch COPD patients.

**Methods:**

The 12 items of the PIH, scored on a self-rated 9-point Likert scale, are used to calculate total and subscale scores (knowledge; coping; recognition and management of symptoms; and adherence to treatment). We used forward-backward translation of the latest version of the Australian PIH to define a Dutch PIH (PIH(Du)). Mokken Scale Analysis and common Factor Analysis were performed on data from a Dutch COPD sample to investigate the psychometric properties of the Dutch PIH; and to determine whether the four-subscale solution previously found for the original Australian PIH could be replicated for the Dutch PIH.

**Results:**

Two subscales were found for the Dutch PIH data (n = 118); 1) knowledge and coping; 2) recognition and management of symptoms, adherence to treatment. The correlation between the two Dutch subscales was 0.43. The lower-bound of the reliability of the total scale equalled 0.84. Factor analysis indicated that the first two factors explained a larger percentage of common variance (39.4% and 19.9%) than could be expected when using random data (17.5% and 15.1%).

**Conclusion:**

We recommend using two PIH subscale scores when assessing self-management in Dutch COPD patients. Our results did not support the four-subscale structure as previously reported for the original Australian PIH.

## Introduction

Self-management interventions aim to improve the health behaviour and self-management skills of patients with chronic and complex health conditions in order to improve the physical health and well-being of these patients [1,2]. Problem solving, decision making, resource utilisation, forming patient-provider partnerships, and patient-tailored action planning are essential parts of self-management [2]. As patient self-management skills develop, increased confidence in their own health management becomes a powerful factor in inducing and sustaining behaviours that provide perceived benefits [2,3]. This is especially important in patients with Chronic Obstructive Pulmonary Disease (COPD) who are responsible for their day-to-day disease management [2]. COPD self-management interventions aim to e.g., instil the confidence to recognise COPD exacerbations [1] and to take appropriate actions when COPD symptoms deteriorate. The most recent Cochrane review regarding COPD self-management interventions showed that COPD self-management interventions are associated with improved health-related quality of life (HRQoL), a reduction in the number of hospitalisations, and improved dyspnoea [4]. In COPD patients, assessments have traditionally involved objective parameters (e.g., lung function). More recently, patient-reported outcomes (PROs) have become increasingly popular. Using PROs, it is not only possible to evaluate outcomes such as COPD-specific HRQoL [5] (e.g., St. George’s Respiratory Questionnaire (SGRQ)) [6] and COPD self-efficacy [7], but also perceived health outcomes. Little is known, however, about perceived health outcomes such as self-management behaviour and knowledge in COPD patients.

To facilitate the measurement of self-management behaviour and self-management knowledge of patients with chronic diseases the 12-item Partners in Health scale (PIH) was developed by an Australian research group [8]. The Australian 12-item PIH was intended to provide a first step of assessing a patient’s self-management in developing a collaborative patient-clinician self-management care plan. It was designed to assist patients with chronic and complex conditions in learning how to participate more effectively in the management of their condition and to improve their self-management skills, because previous research indicated that providing coordinated care for people with chronic conditions was predominantly based on their self-management capabilities rather than on the severity and/or complexity of their illness [9]. The Australian 12-item PIH was therefore introduced as a generic self-rated clinical PRO tool suitable for: 1) assessing the effects of self-management interventions in populations with different chronic conditions; 2) comparing populations; and 3) determining changes in patient self-management knowledge and behaviour over time [8]. Subsequently, it was found to be a valid measure of patient competency in relation to the self-management of their chronic conditions [8]. Four subscales were reported based on Principal Component Analysis (PCA): knowledge, coping, recognition and management of symptoms, and adherence to treatment [8]. Hitherto, the Australian PIH has been successfully used to evaluate (self-) management strategies for chronic disease prevention and management [10]. In addition, the PIH has also been used as a screening tool to identify patients who would most benefit from a self-management care plan [11]. The PIH has been translated into Spanish and validated among healthcare users (patients with diabetes, hypertension and cancer) of primary care in Mexico [12]. Three subscales were reported for the Spanish PIH based on exploratory factor analysis (FA) [12].

Having greater insight into COPD patient behaviour and knowledge would facilitate the identification of key COPD self-management skills that could be improved. This could help inform further improvement of patient-tailored COPD self-management interventions and may reduce the high disease burden, hospitalisations and healthcare cost in COPD patients [13,14]. The PIH has, however, not been validated for use in patients with COPD nor has it been validated in the Dutch language. The aim of the current study was, therefore, to assess the construct validity and reliability of a Dutch translation of the latest PIH version in Dutch patients with COPD. More specifically, we assessed the underlying dimensionality of the Dutch PIH using data from a Dutch COPD sample participating in the COPE-III self-management intervention study [15] to determine whether the same four-subscale solution of self-management for the original Australian PIH as proposed by Petkov et al. [8] could be found for the Dutch PIH.

## Materials and Methods

### Measures

#### Partners in Health scale

The original PIH consists of 12 items (PIHv1), scored on a self-rated 9-point Likert scale with 0 indicating the worst and 8 the best possible patient self-management [8]. Both a total sum score and four subscale scores can be calculated for the PIHv1: knowledge (items 1, 2, 4, 8); coping (items 10–12); recognition and management of symptoms (items 6, 7, 9); adherence to treatment (items 3, 5). Reliability (estimated using Cronbach’s Alpha) equalled 0.82 for the total scale [8]. The 12-item PIHv1 is based on six key principles essential for effective self-management that were transformed into 12 items assessing how well persons were self-managing. It was revised by splitting two double-barrelled items into two questions each; for instance emotional and social impacts of the condition(s) became items 10 and 11 in PIHv2. The resulting 14-item PIH version was used clinically for several years and was also included in a RCT aimed at improving patient self-management competencies [16]. After a national project to determine a consensus definition of self-management the 14-item PIH was further revised [17], which allowed the number of items to be reduced and the time to administer and score the tool minimized, balanced against retention of items that were clinically relevant. Therefore, item 5 from PIHv1 (‘arranging and attend appointments’) was changed into item 6 ‘attend appointments’ in PIHv2. Two questions on monitoring and managing symptoms (item 6 and 8) were removed from PIHv1. In addition, an item on ability to access culturally appropriate services was added (item 5). The result was the current 12-item PIHv2 from which the Dutch version was derived. A copy of PIHv2 can be obtained from Flinders University, Australia.

#### Development of the Dutch PIH

For use in a Dutch speaking population the PIHv2 was translated into Dutch then back-translated into English by an independent translator (guidelines Guillemin et al. [18,19]). A Dutch PIHv2 (PIH(Du)) was defined (see S1 Table) and pre-tested in a qualitative evaluation with a small group of Dutch COPD patients who did not participate in the COPE-III self-management study [15], which is an ongoing RCT regarding self-management in COPD patients with comorbidities. Sampling of patients for the qualitative evaluation was continued until saturation of information was achieved. Comments on the wording, layout of the 9-point Likert scale, and issues encountered during the self-administration process were collected using the three-step test interview (TSTI) [20]. Respondents completed the PIH and concurrently verbalised their thoughts (‘think aloud technique’). Subsequently, they answered probes about terms or phrases in the PIH. A predefined cognitive testing protocol [21] was used for this second step. The third step elicited experiences and opinions of patients [20,21]. Non-verbal communications were documented and all verbalisations were audio recorded for further analysis. Data from the TSTI were analysed using content analysis approach [22], in which coding categories are derived directly from the text data.

### Patients

We used baseline data from Dutch COPD patients with comorbidities participating in the COPE-III study for the psychometric analyses [15]. The patient eligibility criteria have been previously described [15] and can be summarised as follows: a clinical diagnosis of COPD [23]; clinically stable at the time of inclusion; at least one clinically relevant comorbidity (ischemic heart disease, heart failure, diabetes, anxiety and/or depression); at least three COPD exacerbations and/or one hospitalisation for respiratory problems in the two years preceding study entry; and adequate Dutch language proficiency. All procedures performed in the current study were in accordance with the ethical standards of the institutional and/or national research committee and with the 1964 Helsinki declaration and its later amendments or comparable ethical standards. The study protocol was approved by the Medical Ethical Committee at Medisch Spectrum Twente and by the Southern Adelaide Clinical Human Research Ethics Committee. The study is registered in the public Australian New Zealand Clinical Trials Registry (ACTRN12612000514808). Written informed consent was obtained from all individual participants prior to participation in this study.

### Statistical analyses

Descriptive statistics were calculated using SPSS v20.0 [24]. Both scale structure and item properties were analysed. The analytic strategy was defined prior to viewing the dataset. Following Paap et al. (2015) [25], we used two complementary statistical methods to evaluate the dimensionality of the PIH(Du): 1) Mokken Scale Analysis (MSA; a non-parametric technique); and 2) common FA.

In recent years, MSA has increased in popularity in health research [26–31]. MSA identifies scales that allow an ordering of individuals on an underlying scale using unweighted sum scores [32,33]. In order to ascertain which items co-vary and form a scale, scalability coefficients are calculated on three levels: item-pairs (*H*_*ij*_), items (*H*_*i*_), and scale (*H*). *H* is based on *H*_*i*_ and reflects the degree to which the scale can be used to reliably order persons on the latent trait using their sum score. A scale is considered acceptable if 0.3≤*H*<0.4, good if 0.4≤*H*<0.5, and strong if *H*≥0.5 [32,33]. MSA can be used in both a confirmatory and exploratory manner. The exploratory procedure follows a bottom-up, iterative approach. First, a start set of items is identified in one of two ways: 1) the item pair with the highest *H*_*ij*_ value is chosen (default), or 2) the researcher specifies the start set manually. Subsequently, the relationship (in terms of *H* coefficients) of each remaining item with the start set is evaluated one item at a time. At each step, the item that maximises *H* is added, but only if a) it has a positive relationship (in terms of *H*_*ij*_) with the set of items in the current scale, and b) adding the item results in an *H*_*i*_ value higher than a predefined user-specified constant *c* (typically 0.3). When no more items can be added, a second subscale is formed. The procedure stops when no items are left, or when no other items can be assigned to subscales anymore. For more detailed information on MSA, we refer to Paap et al. (2013; online supplement [25]). MSA was applied using the R [34] package Mokken [35]. We ran the exploratory analysis several times in a row, each time increasing the lower bound scalability coefficient *c* [33]. The outcomes indicate whether the data set is one-dimensional or multidimensional [33].

We used Parallel Analysis (PA) based on Minimum Rank Factor Analysis (MRFA); this method will be abbreviated as PA-MRFA [36]. MRFA is a common FA method that allows one to find the “most-unidimensional” solution [37]. In PA-MRFA, for each factor the empirical value of the proportion of explained common variance (ECV) is compared to corresponding factors ECV derived from random data [36]. The random data are generated based on the sample size of the real data assuming independence among items [38]. Typically, a large number of random datasets are generated, resulting in a sampling distribution of ECV-values for each factor. To determine the optimal number of factors, for each successive factor the observed ECV can be compared to the mean or the 95^th^ percentile of the sampling distribution associated with the respective factor. We used the software package FACTOR [39] to perform the PA-MRFA analyses. We used the standard configuration for PA-MRFA: 500 random correlation matrices were generated based on “random permutation of sample values” [36]. Usually, it is advised to use polychoric correlation-based common FA in the case of ordinal data (with five or fewer answering categories). Although the PIH items were scored with nine response options (eligible to be treated as continuous), we had to collapse categories for all items prior to the analyses, in order to ensure adequate coverage (at least 10–15 observations per item-category combination). Polychoric correlation based models would, therefore, be more appropriate. However, they are known to be more prone to convergence issues when small sample sizes are involved. It was therefore decided to run two sets of analyses; one based on polychoric correlations and one based on Pearson correlations. The 95^th^ percentile threshold was used for the polychoric analysis and the mean threshold for the Pearson analysis [36]. Since both sets of models converged and resulted in similar factor solutions, we will only report the findings based on the polychoric correlations. An oblique factor rotation (Promin) was used to facilitate interpretation of the factors [40].

## Results

### Qualitative evaluation of the PIH(Du)

Qualitative data were gathered during interviews with four Dutch COPD patients. In general, the instructions were found to be clear and patients indicated that the PIH(Du) was a proper, readable, synoptic, complete and clear instrument. Critical notes were: use of long sentences; information on a time period that fits with the completion of the instrument was lacking; and it could be more COPD-specific. In addition, more specific comments on the individual items and the clarity of wordings were provided for the items 5–12 (see Table 1). Patients’ suggestions for improvements were, for instance, adding a definition of a ‘healthcare professional’ and ‘blood glucoses level’. Other suggestions were: delete ‘culture, value and beliefs’ from item 5 (“You could leave out the last part of this question (culture, values and beliefs)”); add ‘life style’ and rephrase item 9; and split item 12 into different items for the different healthy life styles (e.g., ‘I manage to live a healthy life with no smoking’, ‘I manage to live a healthy life with moderate alcohol use’). The horizontal axis of the 9-point Likert scale was found acceptable and familiar (“This is quite similar to what they ask in connection with the pain threshold”). However, patients also indicated that a PIH(Du) item score of zero (lowest possible self-management) will most likely only be used by patients with an end-stage disease. Suggested improvements for the 9-point Likert scale were using fewer response options and visualising response options (“You could use it like a traffic light”).

**Table 1:** Results of the qualitative evaluation of the 12-item PIH(Du) in four Dutch COPD patients.

Item	Interpretation	Comments (e.g., on clarity of wordings)	Improvements
1: Knowledge of illness	“What I know in general about my health conditions.” “How much you know yourself about your illness.” “What the health reasons are.” “Whether I have lung issues.” “Whether you are well informed about your own health conditions.”	-	-
2: Knowledge of treatment	“Whether I do know what the treatments and medications are for my conditions.” “It is about what I know in general about the medicines I use.” “The treatment with medication changes so quickly. I think, regarding the information about medicines, that it could be done better.”“And I have pointed that out a few times about my treatment.”	-	-
3: Taking prescribed medication	“Just whether to take the medicines and to follow the treatment instructions.”“Regarding those medicines….nothing is ever said about it or how to use it.” “That you take what is prescribed, as has been agreed with your healthcare provider.”	-	-
4: Decision sharing	“In principle, I always take decisions together with my doctor or healthcare provider.” “Actually, I haven’t been informed about that yet, about what’s wrong—or not wrong—with me.” “I don’t know what, what, what…where I always stand.” “I should talk about it with the doctor or healthcare provider then, shouldn’t I?“Whether you take decisions if you do experience symptoms.”	-	-
5: Services fit with culture/value/beliefs	“Because I do occasionally discuss this with my doctor.” “Should I also arrange for a health professional? That‘s what it seems to say.” “That is self-evident that a healthcare provider should adapt to someone with a different cultural background.”	“Yes, and just what does it all mean?” “I don’t understand it very well.” “But this has nothing to do with the kind of healthcare you need, I think.”“The most important thing is that you are able to arrange your healthcare as much as possible yourself.”	“You could leave out the last part of this question (culture, values and beliefs).”
6: Arrange and attend appointments	“Then you need to go to a doctor or health professional.” “An appointment where I need to go.”	“I’ve never had contact with a health professional. Then I don’t know what this health professional is supposed to do.” “What do you mean by that, a health professional?” “So I’d think this word [health professional] is not appropriate in this questionnaire.	“Add a definition of health professional.”
7: Track of symptoms	“I understand my symptoms.” “Then you need to indicate how and what then. The same goes for your medicines. If I’m breathless or something.” “To act in time if you are not feeling well.” “That you need to know your body well yourself.”“I recognise the symptoms, but I don’t take action.”	“I think that this is a good question.” “This is a very long sentence.” “This is not applicable to me, but I do understand it.” “I cannot fill in fairly well or very well, since I don’t know what that is: peak flow.” “Peak flow? What do they mean by that?” “For instance blood sugar levels and peak flows. I don’t know what that is.” “I don’t know to what extent blood glucose levels, peak flows, weight and sleeping problems are related to COPD. I don’t know that as a layperson, do I?”	“Add a description of peak flow and blood glucoses level to this question.”“Shorten this question.” “Change this question into: ‘For instance, I watch my symptoms or early warning signs, such as breathlessness’, which makes this more relevant for COPD.”
8: Take action when symptoms deteriorate	“Well, then I always tell the doctor when the symptoms get worse.” “Whether I do take action when there are warning signs” “I never take action when I have symptoms or something.” “Yes, well, yes, I do take action. But quite late, usually.” “Usually I contact the pulmonary physician then.”	“Because I also think that many people will not understand this…symptoms and all those kinds of words.”	“If you want to make it easier to understand for everyone, then you could simplify it.” “Make it more concrete.”
9: Dealing with effects on physical activity	“How you function yourself.” “What is possible and what is not possible.” “That I have everything under control, such as performing household chores and walking.” “If I do those activities, how my health will develop.” “If someone leads a regular life, then you will have control over your lungs, over your walking, won’t you.”	“Rather a mouthful, in my opinion. And that question really depends on how your complaints are at that moment.” “Short term or long term?” “Because that depends on how your physical condition is at that moment.” “So I think this question is very difficult defined.” “The effects will come later.” “I think this it is a little bit hard to answer.” “The effect of health conditions, I think that yes, that depends on the severity of your conditions, of course.”	“Maybe add life style.” “So, I would describe it more, like ‘I can control my physical activities such as household chores, walking, in a normal way.’” “And you could put it in an even simpler way, like: ‘I have control over my health conditions and over my daily activities myself. For example, walking and household chores.’”
10: Dealing with effects on emotional wellbeing	“Well, whether I have my emotions under control and that I mentally…That all is well mentally.” “Whether I have control over the effects on my emotional wellbeing.” “Whether I can keep my emotions under control, when I have problems.” “This question is not applicable to me. Actually, I’m always in a good mood.”	“Very long sentences. It’s almost like two questions in one.” [reads first half of question out loud] “(…) the effect of my health condition, I think that is very incomprehensible for many people.” “I think the word ‘effect’ will be filled in differently than what is meant.”	“You need to turn it around. What or with a question: ‘what is the effect of my health…ehm…condition on your own emotions and whether you have it under control?’” “Start this question with ‘I have insight into my health condition’, because that is easier to understand.”
11: Dealing with effects on social life	“I often have things that I think I love to do this or that.” “How I behave and everything.” “Whether I can cope with my health issues.” “I’m not very sociable; I don’t need to be around a lot of people. So I’ll never visit a crowded place.” “It does not have any effect when my symptoms change.”	“Also very broad.” “I think this is more about like a character trait.” “It is a general list. I have trouble relating it to lung problems.”	“Just like before, start this question with ‘I have insight into (…)’.”
12: Manage to live a healthy life	“Whether I am smoking, using alcohol or doing a lot of physical exercise.”	“There are several things incorporated that I think are very difficult to answer.” “It can be difficult to indicate whether you eat healthy, I don’t know that.” “Everything has been added to this question.” “I cannot answer this question by giving one answer, since this question contains different things of a healthy life.”	“Split this question into different questions for the different healthy life styles, e.g., smoking behaviour, alcohol use, sports etc.”

### Patient characteristics

Patient characteristics for the Dutch COPD sample used for psychometric analysis can be found in Table 2. The PIH(Du) (see S2 Table) was completed by 118 COPD patients (65.3% male, mean age 67.6, 19.5% smoker) diagnosed with at least one clinically relevant comorbidity (71.2% cardiovascular disease, 40.7% diabetes, 19.5% anxiety, 16.9% depression).

**Table 2:** Characteristics of Dutch COPD patients with comorbidities who completed the 12-item Dutch Partners in Health scale.

Patient characteristics	Total (n = 118)
age in years; mean (SD)	67.6 (8.9)
male; n (%)	77 (65.3)
smoker; n (%)	23 (19.5)
mMRC dyspnoea score, range 0–4; mean (SD)	1.99 (0.91)
health literacy*, range 1–5; mean (SD)	2.56 (0.92)
lung function parameters; mean (SD)	
FEV_1_% predicted post-bronchodilator	52.4 (14.7)
FEV_1_/FVC post-bronchodilator	51.3 (12.9)
diagnosed disease; n (%)	
COPD	118 (100)
cardiovascular	84 (71.2)
diabetes	48 (40.7)
depression	20 (16.9)
anxiety	23 (19.5)
12-item PIH(Du) total score; mean (SD)	78.1 (9.7)
PIH(Du) subscale 1**; mean (SD)	35.2 (6.9)
PIH(Du) subscale 2***; mean (SD)	42.9 (4.3)

FEV_1_: Forced Expiratory Volume in one second as percent predicted for age, gender and height; FVC: Forced (expiratory) Vital Capacity; mMRC: modified Medical Research Council; PIH(Du): Dutch Partners in Health scale; SD: Standard Deviation

*Health literacy was measured by asking patients for their confidence in completing medical forms by themselves with higher scores indicating lower confidence.

**Subscale 1 was tentatively labelled as ‘knowledge and coping’;

***Subscale 2 was tentatively labelled as ‘recognition and management of symptoms, adherence to treatment’.

### Dimensionality and reliability analyses

Running exploratory MSA indicated a two-dimensional pattern for the PIH(Du) (see Table 3). The two PIH(Du) subscales were tentatively labelled as: 1) knowledge and coping (items 1, 2, 8–12) and 2) recognition and management of symptoms, adherence to treatment (items 3–7). The *H*-values of the two subscales based on the Dutch data were good (0.43, subscale 1) and acceptable (0.38, subscale 2). The correlation between the two subscales was 0.43. The lower-bound of the reliability (estimated using Cronbach’s Alpha) for the total scale equalled 0.84. Cronbach’s Alpha was 0.80 and 0.72 for the PIH(Du) subscales 1 and 2, respectively.

**Table 3:** Scale solutions for the 12-item Dutch Partners in Health scale.

12-item Dutch Partners in Health scale	MSA	PA-MRFA
Item 1: Knowledge of illness	1	1
Item 2: Knowledge of treatment of illness	1	1
Item 3: Taking medication as prescribed	2	2
Item 4: Decision sharing	2	2
Item 5: Services fit with culture/value/beliefs	2	2
Item 6: Arrange and attend appointments	2	2
Item 7: Track of symptoms	2	2
Item 8: Take action when symptoms deteriorate	2	1
Item 9: Dealing with effects on physical activity	1	1
Item 10: Dealing with effects on emotional wellbeing	1	1
Item 11: Dealing with effects on social life	1	1
Item 12: Manage to live a healthy life	1	1

MSA: Mokken Scale Analysis; PA-MRFA: Parallel Analysis based on Minimum Rank Factor Analysis; *Note* The last two columns indicate whether the item was assigned to the Dutch Partners in Health subscale 1 or 2. Subscale 1 was tentatively labelled as ‘knowledge and coping’, subscale 2 was tentatively labelled as ‘recognition and management of symptoms, adherence to treatment’.

The factor analyses resulted in a very similar scale solution to the MSA analyses (see Table 3). The polychoric correlations matrix can be found in Table 4. The first two factors explained a larger percentage of common variance (39.4% and 19.9% for factor 1 and 2, respectively) than could be expected when using random data (see Table 5). The estimated correlation between the factors extracted from the Dutch data was 0.41. The factor analyses for the two PIH(Du) subscales showed that the newly added item 5 showed similar factor loadings for both subscales; 0.39 for subscale 1 and 0.48 for subscale 2 (see Table 6).

**Table 4:** Polychoric correlations matrix for the 12-item Dutch Partners in Health scale.

	**I1**	**I2**	**I3**	**I4**	**I5**	**I6**	**I7**	**I8**	**I9**	**I10**	**I11**	**I12**
**I1**	1.00											
**I2**	0.60	1.00										
**I3**	0.03	0.16	1.00									
**I4**	0.27	0.26	0.73	1.00								
**I5**	0.40	0.38	0.34	0.61	1.0							
**I6**	0.00	0.14	0.70	0.46	0.22	1.00						
**I7**	0.12	0.26	0.42	0.39	0.44	0.20	1.00					
**I8**	0.34	0.31	0.23	0.24	0.50	0.07	0.56	1.00				
**I9**	0.25	0.28	-0.20	-0.05	0.24	-0.04	0.33	0.32	1.00			
**I10**	0.32	0.26	-0.06	0.11	0.40	-0.01	0.22	0.31	0.58	1.00		
**I11**	0.38	0.35	0.20	0.23	0.36	0.21	0.34	0.28	0.47	0.64	1.00	
**I12**	0.20	0.32	0.17	0.23	0.36	0.19	0.34	0.38	0.41	0.60	0.51	1.00

**Table 5:** Results of Minimum Rank Factor Analysis Dutch Partners in Health scale.

Factor	% ECV real data	Mean % ECV random data	95^th^ percentile % ECV random data	Eigenvalue*
1	**39.4**	17.5	20.1	4.17
2	**19.9**	15.1	16.7	2.16
3	9.6	13.4	14.9	0.98
4	8.9	11.8	12.9	0.78
5	6.2	10.3	11.4	0.51
6	5.0	8.9	9.9	0.29
7	3.9	7.5	8.6	0.20
8	3.2	6.1	7.2	0.19
9	2.4	4.6	6.0	0.11
10	0.9	3.2	4.6	0.07
11	0.6	1.8	3.1	0.00
12	0.0	0.0	0.0	0.00

ECV: explained common variance

*Based on reduced correlation matrix

*Note*: Standardized Cronbach’s Alpha (total scale) = 0.84

**Table 6:** Factor loadings of the Dutch Partners in Health scale based on Minimum Rank Factor Analysis.

	PIH(Du) subscale 1: ‘knowledge and coping’	PIH(Du) subscale 2: ‘recognition and management of symptoms, adherence to treatment’
Item 1: Knowledge of illness	**0.57**	0.07
Item 2: Knowledge of treatment of illness	**0.47**	0.19
Item 3: Taking medication as prescribed	-0.39	**1.05**
Item 4: Decision sharing	-0.13	**0.93**
Item 5: Services fit with culture/value/beliefs	0.39	**0.48**
Item 6: Arrange and attend appointments	-0.26	**0.74**
Item 7: Track of symptoms	0.30	**0.45**
Item 8: Take action when symptoms deteriorate	**0.49**	0.26
Item 9: Dealing with effects on physical activity	**0.80**	-0.27
Item 10: Dealing with effects on emotional wellbeing	**0.89**	-0.17
Item 11: Dealing with effects on social life	**0.65**	0.12
Item 12: Manage to live a healthy life	**0.60**	0.13

PIH(Du): Dutch Partners in Health scale. *Note*: To aid interpretation, the factor loadings higher than 0.40 are printed in bold.

## Discussion

Our dimensionality analyses showed a two-subscale solution for the PIH(Du): 1) knowledge and coping; 2) recognition and management of symptoms, adherence to treatment. Our results therefore did not support the four-subscale structure as previously reported for the original Australian PIH [8]. It is of interest that a Spanish version of the PIH was found to have a three-subscale solution [12].

Several possible explanations have been put forward to account for different findings in factorial solutions across studies: differences in statistical methods and target populations, sample size, number of items per factor, number of factors in the model, and the size of the communalities (proportion of the variance of an item that is accounted for by the common factors in the model) [31,41,42]. At the time of the original Australian PIH development [8], its dimensionality was evaluated by using a two-stage procedure: an exploratory PCA (data reduction technique to group items into a set of new variables) and a confirmatory common FA (a mathematical model to estimate the relationship between items and latent variables [43]) was subsequently used to “validate” the structure identified by the exploratory analysis. However, PCA and common FA will only produce similar results under very specific circumstances [38]. We favoured using exploratory IRT and common FA models over PCA in this study, because they are suitable for ordinal data [44] and result in meaningful scales (e.g., Borsboom et al. [45]). It is unclear which exploratory FA was performed for the Spanish PIH validation [12]. We were therefore unable to compare our results with the three-subscale solution for the Spanish PIH.

The MRFA criteria used in our study require less interpretation in determining dimensionality and allows one to find the “most-unidimensional” solution [37], in comparison with conclusions based on a PCA. Petkov et al. used a Cattell’s Scree plot [46] as a graphical representation of the eigenvalues and suggested a cut-off of three components as defined by the ‘elbow’. This choice is somewhat arbitrary and the plot can be interpreted in different ways, since the slope has flattened from two components onwards and, therefore, the cut-off point could also be at two or one component. It has been shown that the Scree test has a tendency to overestimate the number of subscales [47] and it should be used and interpreted with care. Kaiser’s criterion to retain factors with eigenvalues greater than one for interpretation is the best known and most utilised method in practice [48]. Despite its simplicity, though, this method may also lead to arbitrary decisions and be inefficient in determining the number of subscales [48].

There is no consensus about a decision rule for the minimal sample size requirements in dimensionality analyses. In the current study, our sample size of 118 COPD patients is of a small to moderate size, with a correlation between the two PIH(Du) subscales of 0.43 and *H*-values of 0.43 and 0.38. According to the guidelines of Straat et al. (2014) [49] the sample size should be 50 to 250 to obtain 90 to 99% correct item assignment and adequate to good Per Element Accuracy in MSA. For MSA analyses the required minimal sample size is mainly dependent on the correlations between the latent variables and the *H*-values of the items [49]. Based on the correlations and *H*-values we found in the current study, our sample size should be sufficient to obtain 94–99% correct item assignment [49]. For FA the minimally required sample size depends on a complex interplay of many aspects, e.g., the estimated factor loadings and communalities [50]. When communalities are high, sample size tends to have less influence on the quality of factor solutions compared to when communalities are low [50]. In case of relatively low communalities, a larger sample size and number of items per factor are needed to obtain stable results in FA [41]. Conversely, in case of a relatively small sample size, a higher number of items per factor (≥ 4 items per factor [42]), a small number of factors and moderate to high communalities are needed to estimate a model that will give a good representation of the population factors [41]. Since the factorial solutions we found consist of a small number of well-identified factors with moderate to high communalities, we feel confident that our low-dimensional solutions for the PIH(Du) will be easy to replicate.

Cross-cultural differences and adjustments made after publication of the original PIH may also have contributed to the discrepancy in dimensionality between the original Australian PIH and the PIH(Du). For instance, item 5 (‘dealing with health professionals to get services that fit with culture, values and beliefs’), which is unique to the PIHv2, was difficult to interpret for Dutch patients and most patients felt the item was not applicable to them. In addition, item 5 showed high factor loadings on both of the Dutch subscales, making it difficult to assign the item to either scale. We therefore suggest removing this item. Item 10 (‘manage the effect of health condition(s) on emotional wellbeing’) has recently been added by the PIH authors in an attempt to show the psychological/emotional impact of the disease(s). Their clinical experiences so far suggest that the item is powerful in ‘breaking open the case’ to uncover factors that can interfere with self-management. However, this item was poorly-received by patients completing the PIH(Du); patients indicated the item was too lengthy, the formulation too complex and it was unclear what the reference time period was. We therefore suggest specifying a recall period in the PIH.

Differences in heterogeneity between the Australian and Dutch samples may also have contributed to the difference in the number of subscales found. Studies on other self-report instruments, such as the SCL-90, have indicated that the number of dimensions found can be related to for example disease severity [31]. Whereas the original Australian PIH was completed by patients with different kinds of chronic diseases, including respiratory problems, the PIH(Du) was administered exclusively to COPD patients, albeit with comorbidities and different COPD severity scores. Patients may provide different responses if multiple chronic conditions are present. For instance, ‘health condition(s)’, as used in the items 1, 2, 4, 9, 10 and 11 from the PIH(Du) is a broad definition and can be interpreted in different ways. Patients completing the PIH may only have considered those health conditions for which they have recently experienced symptom deterioration. Therefore, when multiple chronic conditions are present, the specific contribution and effects of each chronic condition cannot be assessed by the PIH scores. However, PIH scores were developed to enable assessment of the knowledge and behaviour of patients in general to improve self-management interventions.

Based on our findings, we feel confident that the PIH is a useful tool in assessing self-management behaviour and knowledge in COPD patients, but we do recommend some minor changes to the instrument. Obviously, the PIH requires translation if used in other than the source language, which is often the case in international research [51–53]. However, when, besides translation, other changes are made over time to further improve measurement instruments, this may negatively impact its interpretation for use in research or clinical practice. First, with regard to changes made to the Australian PIH version, clear guidelines are needed before translation and validation of the instrument for use in other settings and countries can be continued. Second, we recommend introducing a recall period. Third, we suggest avoiding the use of terms with multiple meanings and composite items (e.g., it is difficult to respond unequivocally to the question ‘‘I take medications or carry out the treatments” if patients do take their medication, but do not carry out the treatments as asked by the doctor). Furthermore, none of the Dutch patients used all nine response options. Simplifying the PIH by using fewer response options could therefore be considered, although any such change would of course require re-validation.

As a next step in our validation process, we plan to investigate the clinical relevance of the two-subscale solution by assessing the ability of both subscale scores to discriminate between patients who received benefit from the COPD self-management intervention (e.g. better self-treatment adherence, higher quality of life scores, fewer hospitalisations and fewer exacerbation days) and those who did not, and who demonstrated a poor self-management capacity. We will also assess the associations between the subscale scores and e.g. quality of life. In addition, we have planned to assess the responsiveness of the PIH, and whether response shift occurs in COPD patients. A study by Harvey and colleagues showed that self-reported Australian PIH scores improved significantly over time when patients with chronic diseases were involved in peer-led self-management education programs [54]. Their results indicated that patients had improved understanding of their condition and the ability to manage and deal with their symptoms resulting in a positive effect on self-management skills, confidence and health-related behaviour [54]. Our ongoing RCT regarding self-management in COPD patients [15] will allow us to assess the responsiveness of the PIH in more detail.

## Conclusion

This is the first time that a translated Dutch PIH was validated in a sample of Dutch COPD patients. Our findings indicate that most items are well-received by patients and show favourable psychometric properties. We recommend making minor changes and refinements. More importantly, however, there is need for (international) consensus on a final version of the PIH which can be validated in several settings and populations. Nevertheless, the PIH shows great promise to facilitate the identification of self-management skills needing improvement in COPD patients with other comorbid conditions. PIH scores could be used to tailor COPD self-management interventions to the patient’s needs and capabilities, facilitating appropriate self-management of COPD exacerbations and a reduction of hospitalisations. For use in Dutch COPD patients, we recommend using two PIH subscale scores when assessing self-management knowledge and behaviour. More research is needed to evaluate whether this two-subscale solution is optimal for other populations as well.

## Supporting Information

S1 Table Dutch translated 12-item Partners in Health scale (PIH(Du)). (PDF)

S2 Table Observed scores of the Dutch 12-item Partners in Health scale (PIH(Du)). (PDF)

## References

[1] Bourbeau J, van der Palen J. Promoting effective self-management programmes to improve COPD. Eur Respir J 2009 3;33(3):461–3. 10.1183/09031936.00001309 19251792

[2] Lorig KR, Holman H. Self-management education: history, definition, outcomes, and mechanisms. Ann Behav Med 2003 8;26(1):1–7. 12867348 10.1207/S15324796ABM2601_01

[3] Bourbeau J, Nault D, Dang-Tan T. Self-management and behaviour modification in COPD. Patient Educ Couns 2004 3;52(3):271–7. 14998597 10.1016/S0738-3991(03)00102-2

[4] Zwerink M, Brusse-Keizer M, van der Valk PD, Zielhuis GA, Monninkhof EM, van der Palen J, et al Self management for patients with chronic obstructive pulmonary disease. Cochrane Database Syst Rev 2014 3;19;3:CD002990. 10.1002/14651858.CD002990.pub3 PMC7004246 24665053

[5] Guyatt GH, Berman LB, Townsend M, Pugsley SO, Chambers LW. A measure of quality of life for clinical trials in chronic lung disease. Thorax 1987 10;42(10):773–8. 3321537 10.1136/thx.42.10.773 PMC460950

[6] Jones PW, Quirk FH, Baveystock CM, Littlejohns P. A self-complete measure of health status for chronic airflow limitation. The St. George's Respiratory Questionnaire. Am Rev Respir Dis 1992 6;145(6):1321–7. 1595997 10.1164/ajrccm/145.6.1321

[7] Wigal JK, Creer TL, Kotses H. The COPD Self-Efficacy Scale. Chest 1991 5;99(5):1193–6. 2019177 10.1378/chest.99.5.1193

[8] Petkov J, Harvey P, Battersby M. The internal consistency and construct validity of the partners in health scale: validation of a patient rated chronic condition self-management measure. Qual Life Res 2010 9;19(7):1079–85. 10.1007/s11136-010-9661-1 20437206

[9] Battersby M, Harvey P, Mills PD, Kalucy E, Pols RG, Frith PA, et al SA HealthPlus: a controlled trial of a statewide application of a generic model of chronic illness care. Milbank Q 2007;85(1):37–67. 17319806 10.1111/j.1468-0009.2007.00476.x PMC2690310

[10] Walters J, Cameron-Tucker H, Wills K, Schüz N, Scott J, Robinson A, et al Effects of telephone health mentoring in community-recruited chronic obstructive pulmonary disease on self-management capacity, quality of life and psychological morbidity: a randomised controlled trial. BMJ Open 2013 9 6;3(9):e003097 10.1136/bmjopen-2013-003097 24014482 PMC3773640

[11] Battersby M, Harris M, Smith D, Reed R, Woodman R. A pragmatic randomized controlled trial of the Flinders Program of chronic condition management in community health care services. Patient Educ Couns 2015. 10.1016/j.pec.2015.06.003 26146240

[12] Peñarrieta-de Córdova I, Barrios FF, Gutierrez-Gomes T, Piñonez-Martinez Mdel S, Quintero-Valle LM, Castaneda-Hidalgo H. Self-management in chronic conditions: partners in health scale instrument validation. Nurs Manag (Harrow) 2014 3;20(10):32–7. 24571163 10.7748/nm2014.02.20.10.32.e1084

[13] Effing T, Kerstjens H, van der Valk, Zielhuis G, van der Palen J. (Cost)-effectiveness of self-treatment of exacerbations on the severity of exacerbations in patients with COPD: the COPE II study. Thorax 2009 11;64(11):956–62. 10.1136/thx.2008.112243 19736179

[14] Rice KL, Dewan N, Bloomfield HE, Grill J, Schult TM, Nelson DB, et al Disease Management Program for Chronic Obstructive Pulmonary Disease: A Randomized Controlled Trial. Am J Respir Crit Care Med 2010 1 14. 10.1164/rccm.200910-1579OC 20075385

[15] Lenferink A, Frith P, van der Valk P, Buckman J, Sladek R, Cafarella P, et al A self-management approach using self-initiated action plans for symptoms with ongoing nurse support in patients with Chronic Obstructive Pulmonary Disease (COPD) and comorbidities: The COPE-III study protocol. Contemp Clin Trials 2013 9;36(1):81–9. 10.1016/j.cct.2013.06.003 23770110

[16] Battersby MW, Harris M, Reed RL, Harvey PW, Woodman R.J., Frith P. A randomised trial of the Flinders Program to improve patient self-management compentencies in a range of chronic conditions: study rationale and protocol. Australasian Medical Journal AMJ 2010;1(3):198–204.

[17] Battersby M, Lawn S. Capabilities for supporting prevention and chronic condition self-management: A resource for educators of primary health care professionals. Flinders Human Behaviour and Health Research Unit, Flinders University, Bedford Park, SA; 2009.

[18] Guillemin F, Bombardier C, Beaton D. Cross-cultural adaptation of health-related quality of life measures: literature review and proposed guidelines. J Clin Epidemiol 1993;46(12):1417–32. 8263569 10.1016/0895-4356(93)90142-n

[19] Guillemin F. Cross-cultural adaptation and validation of health status measures. Scand J Rheumatol 1995;24(2):61–3. 7747144 10.3109/03009749509099285

[20] Hak T, van der Veer K, Jansen H. The Three-Step Test-Interview (TSTI): An observation-based method for pretesting self-completion questionnaires. Journal of the European Survey Research Association 2008;2(3):143–50.

[21] Willis GB. Cognitive interviewing: A tool for improving questionnaire design. Thousand Oaks (CA): SAGE; 2005.

[22] Hsieh HF, Shannon SE. Three approaches to qualitative content analysis. Qual Health Res 2005 11;15(9):1277–88. 16204405 10.1177/1049732305276687

[23] Rabe KF, Hurd S, Anzueto A, Barnes PJ, Buist SA, Calverley P, et al Global strategy for the diagnosis, management, and prevention of chronic obstructive pulmonary disease: GOLD executive summary. Am J Respir Crit Care Med 2007 9 15;176(6):532–55. 17507545 10.1164/rccm.200703-456SO

[24] IBM SPSS Statistics for Windows, Version 20.0. Armonk, NY: IBM Corp. [computer program]. Version 20.0. Armonk, NY: IBM Corp.; 2011.

[25] Paap MCS, Brouwer D, Glas CAW, Monninkhof EM, Forstreuter B, Pieterse ME, et al The St George's Respiratory Questionnaire revisited: a psychometric evaluation. Qual Life Res 2015. 10.1007/s11136-013-0570-y 24241770

[26] Watson R, van der Ark LA, Lin LC, Fieo R, Deary IJ, Meijer RR. Item response theory: how Mokken scaling can be used in clinical practice. J Clin Nurs 2012 10;21(19–20):2736–46. 10.1111/j.1365-2702.2011.03893.x 21883577

[27] Sijtsma K, Emons WH, Bouwmeester S, Nyklicek I, Roorda LD. Nonparametric IRT analysis of Quality-of-Life Scales and its application to the World Health Organization Quality-of-Life Scale (WHOQOL-Bref). Qual Life Res 2008 3;17(2):275–90. 10.1007/s11136-007-9281-6 18246447 PMC2238782

[28] Sutterlin S, Paap MCS, Babic S, Kubler A, Vogele C. Rumination and age: some things get better. J Aging Res 2012. 10.1155/2012/267327 PMC3303571 22500227

[29] Beukers F, Houtzager BA, Paap MCS, Middelburg KJ, Hadders-Algra M, Bos AF, et al Parental psychological distress and anxiety after a successful IVF/ICSI procedure with and without preimplantation genetic screening: follow-up of a randomised controlled trial. Early Hum Dev 2012 9;88(9):725–30. 22460061 10.1016/j.earlhumdev.2012.03.001

[30] Paap MCS, Kreukels BP, Cohen-Kettenis PT, Richter-Appelt H, de Cuypere G, Haraldsen IR. Assessing the utility of diagnostic criteria: a multisite study on gender identity disorder. J Sex Med 2011 1;8(1):180–90. 10.1111/j.1743-6109.2010.02066.x 20946149

[31] Paap MCS, Meijer RR, Cohen-Kettenis PT, Richter-Appelt H, de Cuypere G, Kreukels BP, et al Why the factorial structure of the SCL-90-R is unstable: comparing patient groups with different levels of psychological distress using Mokken Scale Analysis. Psychiatry Res 2012 12 30;200(2–3):819–26. 10.1016/j.psychres.2012.03.012 22494703

[32] Mokken RJ. A theory and procedure of scale analysis. The Hague: Mouton; 1971.

[33] Sijtsma K, Molenaar IW. Introduction to Nonparametric Item Response Theory. Thousand Oaks: Sage Publications 2002;5.

[34] R: A language and environment for statistical computing [computer program]. Vienna: R Foundation for Statistical Computing; 2013.

[35] van der Ark LA. Mokken scale analysis in R. Journal of Statistical Software, 2007;20(11):1–19.

[36] Timmerman ME, Lorenzo-Seva U. Dimensionality assessment of ordered polytomous items with parallel analysis. Psychol Methods 2011 6;16(2):209–20. 10.1037/a0023353 21500916

[37] Ten Berge JMF, Sočan G. The greatest lower bound to the reliability of a test and the hypothesis of unidimensionality. Psychometrika 2004;69(4):613–25.

[38] Timmerman ME, Lorenzo-Seva U, Ceulemans E. The number of factors problem In: Irwing P, Booth T, Hughes DJ, editors. The Wiley-Blackwell Handbook of Psychometric Testing.Oxford: Wiley-Blackwell; 2015.

[39] Lorenzo-Seva U, Ferrando PJ. FACTOR: a computer program to fit the exploratory factor analysis model. Behav Res Methods 2006 2;38(1):88–91. 16817517 10.3758/bf03192753

[40] Lorenzo-Seva U. Promin: A Method for Oblique Factor Rotation. Multivariate Behavioral Research 1999;34(3):347–65.

[41] MacCallum R, Widaman KF, Zhang S, Hong S. Sample size in factor analysis. Psychological Methods 1999;4(1):84–99.

[42] Marsh H, Hau K, Balla J, Grayson D. Is more ever too much? The number of indicators per factor in confirmatory factor analysis. Multivariate Behavioral Research 1998;33(2):181–220. 10.1207/s15327906mbr3302_1 26771883

[43] Field A. Exploratory factor analysis Discovering statistics using SPSS. 3 ed SAGE Publications Inc.; 2009 p. 627–85.

[44] Wismeijer AA, Sijtsma K, van Assen MA, Vingerhoets AJ. A comparative study of the dimensionality of the self-concealment scale using principal components analysis and Mokken scale analysis. J Pers Assess 2008 7;90(4):323–34. 10.1080/00223890802107875 18584441

[45] Borsboom D. The attack of the psychometricians. Psychometrika 2006 9;71(3):425–40. 19946599 10.1007/s11336-006-1447-6 PMC2779444

[46] Cattell R. The scree test for the number of factors. Multivariate Behavioral Research 1966;(1):245–76. 26828106 10.1207/s15327906mbr0102_10

[47] Zwick W, Velicer W. Comparison of five rules for determining the number of components to retain. Psychological Bulletin 1986;99:432–42.

[48] Fabrigar L, Wegener D, MacCallum R, Strahan E. Evaluating the use of exploratory factor analysis in psychological research. Psychologicial Methods 1999;3:272–99.

[49] Straat JH, van der Ark LA, Sijtsma K. Minimum Sample Size Requirements for Mokken Scale Analysis. Educational and Psychological Measurement 2014;74(5):809–22.

[50] Hogarty KY, Hines CV, Kromrey JD, Ferron JM, Mumford KR. The quality of factor solutions in exploratory factor analysis: The influence of sample size, communality, and overdetermination. Educational and Psychological Measurement 2005;65(202):226.

[51] Beaton DE, Bombardier C, Guillemin F, Ferraz MB. Guidelines for the process of cross-cultural adaptation of self-report measures. Spine (Phila Pa 1976; %2000 12 15;25(24):3186–91. 11124735 10.1097/00007632-200012150-00014

[52] Bullinger M, Alonso J, Apolone G, Leplege A, Sullivan M, Wood-Dauphinee S, et al Translating health status questionnaires and evaluating their quality: the IQOLA Project approach. International Quality of Life Assessment. J Clin Epidemiol 1998 11;51(11):913–23. 9817108 10.1016/s0895-4356(98)00082-1

[53] Wiesinger GF, Nuhr M, Quittan M, Ebenbichler G, Wolfl G, Fialka-Moser V. Cross-cultural adaptation of the Roland-Morris questionnaire for German-speaking patients with low back pain. Spine (Phila Pa 1976;1999 6 1;24(11):1099–103. 10361659 10.1097/00007632-199906010-00009

[54] Harvey PW, Petkov JN, Misan G, Fuller J, Battersby MW, Cayetano TN, et al Self-management support and training for patients with chronic and complex conditions improves health-related behaviour and health outcomes. Aust Health Rev 2008 5;32(2):330–8. 18447824 10.1071/ah080330

