# Supplementary material for: Construct Validity of the Dutch Version of the 12-Item Partners in Health Scale: Measuring Patient Self-Management Behaviour and Knowledge in Patients with Chronic Obstructive Pulmonary Disease
Source: PLoS One. 2016 Aug 26;11(8):e0161595. doi: 10.1371/journal.pone.0161595 (PMC5001637; doi:10.1371/journal.pone.0161595)
Supplement: S1 Table — (PDF) [file pone.0161595.s001.pdf]

S1 Table. Dutch translated 12-item Partners in Health scale (PIH(Du))

Item	Original Partners in Health scale	Dutch translation Partners in Health scale
1	Overall, what I know about my health condition(s) is:	Wat ik globaal weet over mijn gezondheidsaandoening(en) is
2	Overall, what I know about the treatment, including medications of my health condition(s) is	Wat ik globaal weet over de behandeling en medicijnen van mijn aandoening(en) is
3	I take medications or carry out the treatments asked by my doctor or health worker	Ik neem de door de arts of zorgverlener voorgeschreven medicijnen of volg de behandeling
4	I share in decisions made about my health condition(s) with my doctor or health worker	Beslissingen betreffende mijn gezondheidsaandoening(en) neem ik samen met mijn arts of zorgverlener
5	I am able to deal with health professionals to get the services I need that fit with my culture, values and beliefs	Ik ben in staat om met gezondheidsdeskundigen te regelen dat ik de zorg krijg die ik nodig heb en die past bij mijn cultuur, waarden en overtuigingen
6	I attend appointments as asked by my doctor or health worker	Ik ga naar de door de arts of gezondheidswerker verzochte afspraken:
7	I keep track of my symptoms and early warning signs (e.g. blood sugar levels, peak flow, weight, shortness of breath, pain, sleep problems, mood)	Ik houd mijn symptomen en vroege waarschuwingstekens in de gaten (bijv. bloedsuikerwaarden, piekwaarde, gewicht, kortademigheid, pijn, slaapproblemen, gemoedstoestand)
8	I take action when my early warning signs and symptoms get worse	Ik grijp in als de waarschuwingstekens en symptomen verergeren
9	I manage the effect of my health condition(s) on *my physical activity* (i.e. walking, household tasks)	Ik heb het effect van mijn gezondheidsaandoening(en) op *mijn fysieke activiteit* onder controle (d.w.z. lopen, huishoudelijke taken)
10	I manage the effect of my health condition(s) on *how I feel* (i.e. my emotions and spiritual wellbeing)	Ik heb het effect van mijn gezondheidsaandoening(en) op *hoe ik me voel* onder controle (d.w.z. mijn emoties en geestelijk welzijn)
11	I manage the effect of my health condition(s) on *my social life* (i.e. how I mix with other people)	Ik heb het effect van mijn gezondheidsaandoening(en) op *mijn sociale leven* onder controle (d.w.z. hoe ik met andere mensen om ga)
12	Overall, I manage to live a healthy life (e.g. no smoking, moderate alcohol, healthy food, regular physical activity, manage stress)	In het algemeen lukt het mij een gezond leven te leiden (bijv. niet roken, matig alcoholgebruik, gezond eten, regelmatig bewegen, omgaan met stress)

©The Partners in Health Scale is the Intellectual Property of Flinders University and is under copyright. For permission to use the PIH please contact Prof Malcolm Battersby, email: ccm@flinders.edu.au
