# Supplementary material for: Construct Validity of the Dutch Version of the 12-Item Partners in Health Scale: Measuring Patient Self-Management Behaviour and Knowledge in Patients with Chronic Obstructive Pulmonary Disease
Source: PLoS One. 2016 Aug 26;11(8):e0161595. doi: 10.1371/journal.pone.0161595 (PMC5001637; doi:10.1371/journal.pone.0161595)
Supplement: S2 Table — (PDF) [file pone.0161595.s002.pdf]

S2 Table. Observed scores of the Dutch 12-item Partners in Health scale (PIH(Du))

RANDnr	PiH1	PiH2	PiH3	PiH4	PiH5	PiH6	PiH7	PiH8	PiH9	PiH10	PiH11	PiH12
**1**	8	7	7	8	7	8	7	8	6	8	8	8
**2**	8	8	7	7	8	8	8	7	7	7	7	7
**3**	6	6	8	6	0	8	7	7	6	8	8	5
**4**	7	7	8	7	4	8	7	7	4	4	6	7
**5**	6	6	8	8	7	8	7	6	7	8	7	7
**6**	4	5	8	8	4	8	5	4	4	5	5	6
**7**	6	7	8	8	7	8	8	8	6	8	8	6
**8**	5	4	8	6	6	8	5	6	4	5	4	7
**9**	6	6	8	7	6	8	6	7	3	7	4	5
**10**	3	3	3	4	4	4	8	4	8	5	3	3
**11**	5	6	8	7	6	8	6	6	6	4	5	7
**12**	7	7	8	8	8	8	8	8	7	7	7	8
**13**	4	6	8	8	7	8	7	7	4	6	6	6
**14**	4	8	8	7	7	8	8	8	4	4	8	8
**15**	7	7	7	7	7	8	7	6	6	5	7	3
**16**	7	7	8	8	7	8	8	8	6	7	7	7
**17**	6	5	8	8	7	8	8	6	2	7	7	6
**18**	7	7	7	7	7	7	7	7	7	7	5	7
**19**	8	8	8	8	8	8	4	8	4	8	4	8
**20**	7	6	8	6	7	4	6	7	4	7	8	6
**21**	8	8	8	8	8	8	8	8	4	4	4	0
**22**	7	7	8	7	6	8	8	8	5	4	4	4
**23**	7	7	8	8	8	8	6	6	7	8	8	5
**24**	5	5	5	5	4	7	7	7	5	7	7	7
**25**	5	6	8	8	6	8	7	7	5	5	6	6
**26**	6	5	8	8	8	8	8	6	6	8	8	8
**28**	8	8	8	8	8	8	8	8	8	8	8	8
**29**	6	7	8	8	8	8	8	7	6	6	7	6
**30**	4	4	8	8	0	8	4	4	4	0	4	4

**31**	8	6	8	8	8	8	6	8	6	8	8	6
**32**	4	8	8	8	8	8	8	8	4	4	7	5
**33**	8	8	8	8	8	8	8	7	3	7	7	7
**34**	6	6	8	7	7	6	8	8	0	3	4	4
**35**	6	6	8	8	8	8	7	6	6	7	7	6
**36**	8	6	6	6	7	7	6	8	7	6	6	3
**37**	7	7	8	7	8	8	7	8	7	7	8	8
**38**	6	7	8	7	7	8	7	8	5	4	4	4
**39**	5	5	6	6	6	8	6	7	6	7	6	7
**40**	4	5	7	7	4	6	5	6	4	6	6	4
**41**	4	4	8	8	6	8	8	6	5	7	6	6
**42**	6	5	8	8	7	8	8	7	6	7	8	7
**43**	6	6	8	7	8	8	7	8	6	6	6	6
**44**	4	6	8	8	7	8	8	7	7	7	7	7
**45**	7	7	7	7	8	8	8	8	7	8	8	8
**46**	7	7	8	7	7	8	8	8	8	8	8	7
**47**	7	7	8	7	4	8	6	6	5	5	5	1
**48**	6	6	7	7	7	8	7	8	7	7	7	5
**49**	8	8	8	8	8	8	8	8	8	8	8	8
**50**	5	5	8	8	7	8	8	8	5	5	4	5
**51**	6	5	7	6	7	7	7	6	6	5	6	5
**52**	4	5	5	6	4	7	6	7	5	5	7	7
**53**	4	4	8	8	8	8	8	8	4	4	4	4
**55**	6	6	7	5	5	8	6	6	6	6	7	3
**56**	7	5	7	2	2	8	7	8	6	6	6	7
**57**	4	4	8	8	7	8	8	6	4	3	4	0
**58**	5	5	8	6	6	8	8	7	5	6	6	7
**59**	0	4	8	8	8	8	8	8	4	4	4	8
**60**	7	8	7	8	7	7	7	7	0	0	4	0
**61**	7	7	8	8	7	8	7	7	7	6	7	7

**62**	5	5	7	7	7	7	7	7	5	5	3	5
**63**	7	7	8	7	7	8	7	8	6	6	7	6
**64**	6	4	8	8	8	8	6	7	4	5	5	5
**65**	7	7	8	8	8	8	6	6	7	6	6	6
**66**	7	7	8	8	1	8	7	7	4	4	7	7
**67**	6	6	7	7	7	7	7	7	6	6	6	7
**68**	2	5	7	6	7	8	2	7	6	6	5	6
**69**	6	7	7	7	7	7	8	8	7	7	6	7
**71**	7	7	8	8	8	8	8	8	6	6	7	7
**72**	4	4	6	6	6	8	7	7	6	7	7	4
**73**	5	6	7	7	6	8	6	6	5	6	6	4
**75**	6	6	8	4	7	8	5	6	2	6	7	4
**76**	6	8	8	8	8	8	8	8	8	8	7	6
**77**	7	7	8	8	4	8	7	7	3	4	6	2
**78**	7	7	8	8	8	8	7	8	7	7	7	8
**79**	6	6	6	6	6	8	6	6	4	8	5	8
**80**	7	7	8	8	8	8	8	8	8	8	7	8
**81**	6	6	8	8	7	8	7	7	4	2	6	6
**82**	5	6	8	7	6	6	6	7	5	6	6	7
**83**	8	8	6	8	8	8	4	6	5	4	5	3
**84**	6	7	8	4	7	8	7	8	3	6	6	3
**85**	6	6	7	7	7	8	7	8	6	6	6	7
**86**	8	4	6	8	8	6	0	8	0	7	0	0
**87**	7	7	8	8	8	8	7	7	6	7	6	7
**90**	8	7	8	8	7	8	7	8	8	8	8	8
**91**	4	4	8	8	8	8	6	7	3	3	6	7
**92**	6	5	8	8	7	8	6	7	5	5	6	3
**93**	5	4	8	8	8	8	8	8	5	8	8	8
**94**	6	6	8	8	6	8	7	7	6	6	7	5
**95**	6	6	8	8	8	8	8	8	7	8	8	8

**96**	6	6	8	7	7	8	7	5	0	6	7	7
**97**	7	7	7	7	8	5	7	7	6	8	7	7
**98**	6	6	7	4	5	8	3	3	5	4	5	4
**99**	6	6	7	6	7	8	7	7	6	4	4	6
**100**	6	7	8	8	8	8	7	8	7	7	8	7
**101**	8	4	8	8	8	8	8	8	4	4	8	4
**102**	4	0	8	0	0	8	8	8	8	4	4	4
**103**	6	7	7	7	7	8	7	7	7	7	7	5
**104**	6	6	7	4	1	8	5	7	7	6	7	7
**105**	6	7	7	7	8	8	7	7	7	6	6	5
**106**	4	0	8	7	7	8	7	7	4	3	4	4
**107**	8	8	8	8	8	8	8	4	4	4	8	4
**109**	6	6	7	7	8	8	8	8	8	8	7	8
**110**	7	7	8	8	8	8	8	8	7	7	7	8
**112**	8	6	8	8	8	8	8	8	8	7	8	7
**114**	5	6	8	8	8	8	6	8	5	5	7	5
**115**	8	8	8	8	8	7	8	8	8	8	8	8
**116**	6	5	7	7	7	7	6	7	5	6	6	4
**117**	6	6	6	7	7	7	6	7	7	5	7	6
**118**	5	5	8	8	7	8	5	5	3	3	3	4
**119**	7	8	8	8	8	8	8	8	7	8	8	8
**121**	7	5	7	6	4	8	6	4	5	3	5	6
**122**	5	6	8	8	7	8	8	7	7	6	7	7
**122**	7	7	8	8	7	8	8	8	1	1	1	1
**123**	5	6	8	8	7	8	6	7	6	6	7	5
**124**	5	4	8	8	4	8	6	6	4	4	4	4
**125**	6	6	7	7	7	7	7	7	7	7	7	5
**127**	6	6	8	8	8	8	8	8	7	7	8	7
**128**	5	6	7	6	7	8	6	7	7	7	7	2

RANDnr = randomisation number (person index), n=118 cases.

Note: observed item scores before collapsing categories
